# Case report of three cases and literature review: is follicular occlusion triad with peripheral/axial spondyloarthritis involvement an analog of SAPHO syndrome?

**DOI:** 10.3389/fmed.2026.1876061

**Published:** 2026-08-24

**Authors:** Dongdong Zhao, Jinwan Du, Ran Man, Shaohui Geng, Shike Shang, Chen Li

**Affiliations:** 1 School of Life Science, Beijing University of Chinese Medicine, Beijing, China; 2 Liangjiang Hospital of Chongqing Medical University (People’s Hospital of Chongqing Liangjiang New Area), Chongqing, China; 3 School of Traditional Chinese Medicine, Beijing University of Chinese Medicine, Beijing, China; 4 Yueyang Hospital of Integrated Traditional Chinese and Western Medicine, Shanghai University of Traditional Chinese Medicine, Shanghai, China; 5 Shanghai Guanghua Hospital of Integrated Traditional Chinese and Western Medicine, Shanghai University of Traditional Chinese Medicine, Shanghai, China

**Keywords:** disease pattern, follicular occlusion triad, SAPHO syndrome, spondyloarthritis, treatment response

## Abstract

**Background:**

SAPHO syndrome is a rare chronic rheumatic disease characterized by five typical manifestations: synovitis, acne, pustulosis, hyperostosis, and osteitis. The follicular occlusion triad (FOT) comprises hidradenitis suppurativa (HS), acne conglobata (AC), and dissecting cellulitis of the scalp (DCS). The associations of FOT with peripheral/axial spondyloarthritis remain poorly understood. This study aimed to delineate the pattern of possible peripheral/axial spondyloarthritis involvement in patients with FOT.

**Methods:**

All cases of FOT with peripheral/axial spondyloarthritis treated at our center were reported and compared with previously reported cases from the literature. Epidemiological and clinical data were collected from these cases.

**Results:**

Three cases of FOT with peripheral/axial spondyloarthritis from our center and 10 cases from the literature review were included. We confirmed the FOT diagnosis in these patients and analyzed their associated spondyloarthritis involvement. The clinical features and treatments of these cases were also summarized. Most patients were male (92.31%, 12/13). In all patients, skin lesions preceded the onset of spinal arthritis, with intervals ranging from 0.6 to over 15 years. Symptoms related to axial arthritis typically presented as cervical and/or lumbar pain and stiffness (46.15%, 6/13). Only a few patients exhibited the typical sternoclavicular involvement seen in SAPHO syndrome (15.38%, 2/13). Laboratory examinations revealed elevated inflammatory markers and negative rheumatoid factor/HLA-B27 in all patients. Prednisone and adalimumab were reported to provide prolonged remission, while one patient responded satisfactorily to isotretinoin treatment.

**Conclusion:**

We provide an updated overview of the disease pattern of peripheral/axial spondyloarthritis involvement in patients with FOT.

## Introduction

Synovitis, acne, pustulosis, hyperostosis, and osteitis (SAPHO) syndrome mainly includes four different clinical phenotypes: bone and joint involvement associated with palmoplantar pustulosis (PPP) and severe acne (SA); bone and joint involvement with nail changes; hyperostosis with or without dermatosis; and chronic recurrent multifocal osteomyelitis (CRMO) with or without dermatosis (1, 2). In our previous studies, bone and joint involvement in SAPHO syndrome is characterized by the coexistence of osteolysis and osteosclerosis, primarily in the chest wall and spine (3–5). The follicular occlusion triad (FOT) was first reported by Chicarilli in (6). In our previous studies, bone and joint involvement in SAPHO syndrome is characterized by the coexistence of osteolysis and osteosclerosis, primarily in the chest wall and spine. It is composed of hidradenitis suppurativa (HS), acne conglobata (AC), and dissecting cellulitis of the scalp (DCS), and follows a course of chronic relapse and remission, accompanied by inflammation and infectious nodule attacks. The association of FOT with musculoskeletal symptoms has rarely been reported, except in isolated cases or limited case series. FOT and SAPHO syndrome share overlapping cutaneous features, and FOT has been reported to be associated with spondyloarthritis (SpA) (7). However, cases of FOT associated with SpA or SAPHO syndrome have not been extensively investigated. To complicate matters further, the relationship between SpA and SAPHO syndrome remains unclearly defined. The two diseases share a variety of common musculoskeletal features, including sacroiliitis and enthesitis (8, 9). In a survey conducted by Furer et al., nearly half of the physicians considered SAPHO syndrome a subtype of SpA, while 25.6% considered SAPHO a separate entity. Therefore, it is of considerable interest whether patients with FOT diagnosed as having SAPHO syndrome or SpA exhibit different clinical pictures and should be managed differently.

While FOT and SAPHO share some clinical features, they may be underpinned by distinct pathophysiological mechanisms (10). The core pathological feature of SAPHO syndrome is sterile osteitis, characterized by significantly elevated plasma levels of IL-8 and IL-18 (11). In addition, polymorphonuclear neutrophils (PMNs) in SAPHO patients show an impaired ability to induce TNF-α and IL-8 in response to *Propionibacterium acnes*, suggesting that the pathogenesis involves an abnormal innate immune response to specific pathogens and polygenic autoinflammatory pathways (12). In contrast, the pathogenesis of FOT begins with follicular occlusion, followed by follicular rupture and release of keratin into the dermis. This triggers activation of macrophages and dendritic cells, which release TNF-α and IL-1β, subsequently polarizing Th17 cells to secrete cytokines such as IL-17 and IL-22, thereby forming a chronic inflammatory loop centered on the IL-17/Th17 axis (13). These differences in cytokine profiles and target tissues suggest that FOT-associated SpA may not be a simple phenotypic variant of SAPHO but could represent a distinct disease entity with a different pathophysiological background.

In this study, we reported 3 cases of FOT with SAPHO syndrome from our own center and reviewed the FOT cases in the literature. The aim is to compare the musculoskeletal manifestations, imaging features, and treatment responses between FOT-associated spondyloarthritis and classical SAPHO syndrome, in order to explore whether FOT with spondyloarthritis should be regarded as an analog of SAPHO or as a separate disease category, thereby providing clinical reference for the recognition and classification of this rare comorbidity.

## Case presentation

### Case 1

**Figure 1:**
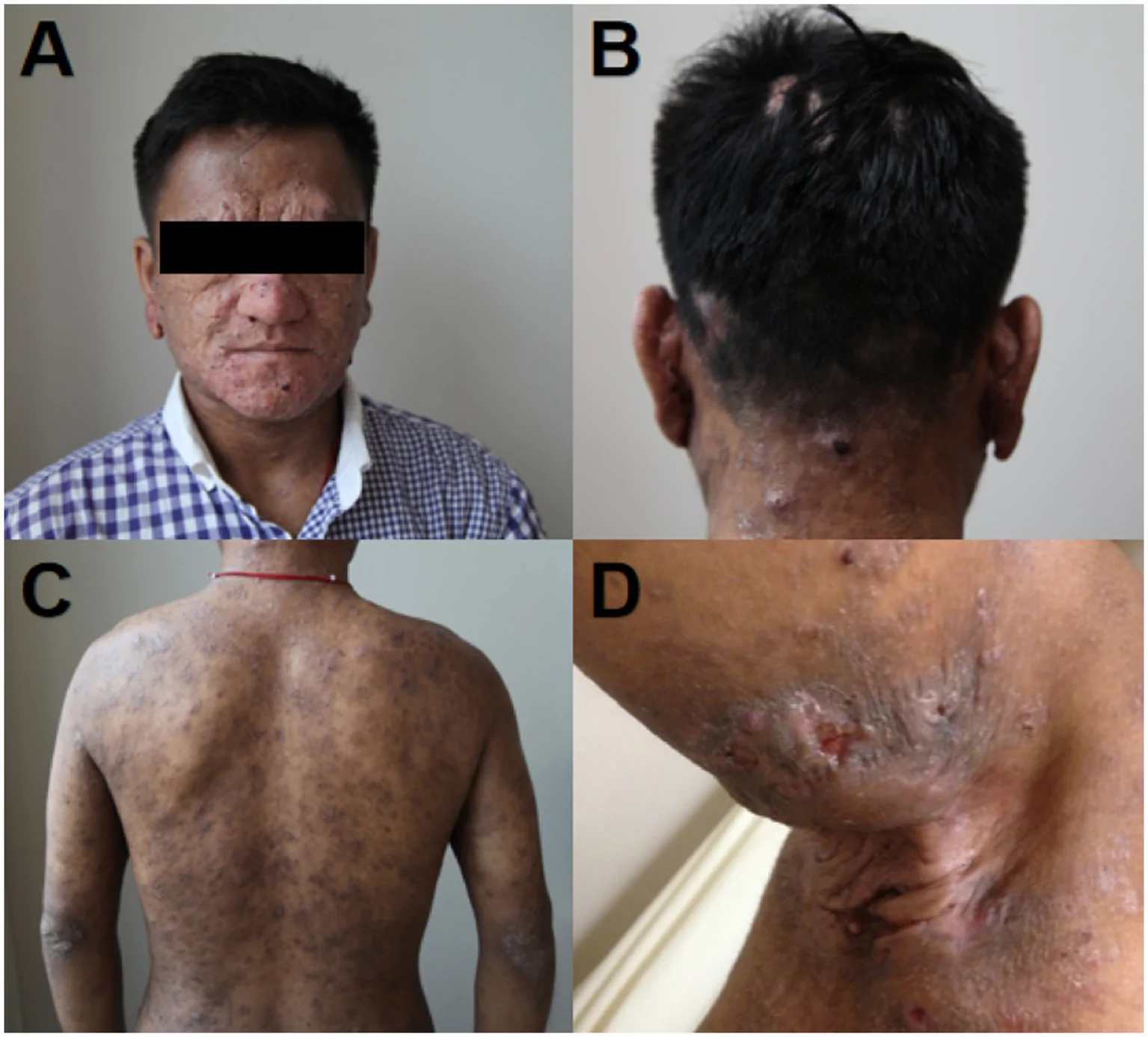
Cutaneous manifestations in Patient No. 1. **(A)** Acne conglobata on the face. **(B)** Confluent atrophic, scarred, erythematous plaques on the scalp. **(C)** Papules on trunk and limbs. **(D)** Hidradenitis suppurativa with fistulas on the armpit.

A 23-year-old male presented with facial acne and skin rash for 5 years. The patient’s facial skin involvement mainly presented as papules, pustules, and cysts (Figure 1A). Multiple draining sinuses were found on his scalp and in his armpit (Figures 1B,D). There were purulent and serosanguinous exudate and scattered papules on his trunk and limbs (Figure 1C). A skin biopsy was performed on a representative lesion from the patient’s back. Histopathological examination revealed epidermal papillomatous hyperplasia, marked hyperkeratosis, and acanthosis, accompanied by dermal perifollicular lymphocytic infiltrates, follicular plugging, and occasional ruptured follicles with granulomatous reaction. His cutaneous manifestations improved slightly with treatment with isotretinoin and minocycline.

**Figure 2:**
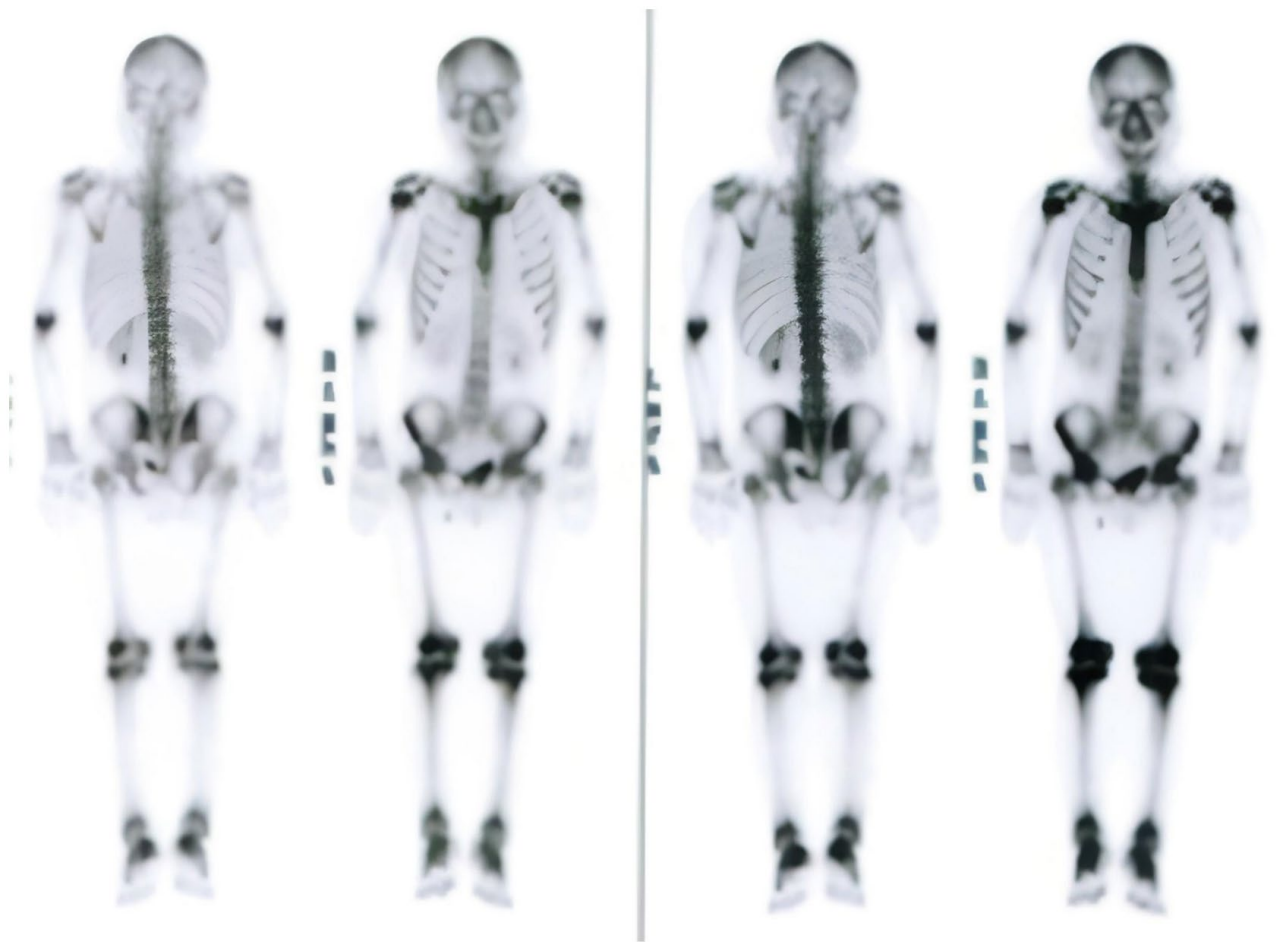
^99m^Tc-MDP whole body bone image of Patient No. 1.

Six months before the patient presented, the patient developed chronic lumbar pain and denied pain in the sternoclavicular joint or limb joints. He denied any history of anemia, tuberculosis, or malignancy. Laboratory tests showed an elevated erythrocyte sedimentation rate (ESR, 100 mm/h) and hypersensitive C-reactive protein (hsCRP, 64.17 mg/L). His antinuclear antibody (ANA), extractable nuclear antigen (ENA), anti-double-stranded DNA antibody (anti-dsDNA), HLA-B27, and rheumatoid factor (RF) results were all negative. ^99m^Tc-methylene diphosphonate (^99m^Tc-MDP) bone scintigraphy showed abnormal tracer uptake in the bilateral shoulders, elbows, knees, and ankle joints (Figure 2).

The patient was diagnosed with FOT complicated by SAPHO syndrome. He was treated with minocycline, cyproheptadine, folic acid, topical sulfacetamide cream, and polysulfide wash. The skin rash improved, and facial papules and cysts partially subsided.

### Case 2

A 50-year-old male presented to the rheumatology department for further evaluation of recurrent acne and polyarthralgia. At age 23, he developed severe acne, pustules, nodules, and pitted scars all over the body, sparing the hands and feet, most severe on the buttocks and in the armpits. A skin biopsy showed hyperkeratosis with dyskeratosis, thickening of the spinous layer, and infiltrating masses composed of neutrophils in the dermis. The clinical presentation and biopsy confirmed the diagnosis of FOT; symptoms slightly improved with minocycline treatment.

At age 41, the patient had suffered pain at the attachment points of the sternoclavicular joints, shoulders, elbows, knees, and heels, and both hands and feet showed local redness, swelling, and pain. ^99m^Tc-MDP whole-body bone imaging showed increased radioactivity in the bilateral sternoclavicular joints, first anterior ribs, shoulder joints, pubic symphysis, left tibia, and right heel. Laboratory tests showed slightly elevated ESR [22 mm/h, reference <15 mm/h (14)] and hsCRP (17.87 mg/L) with negative RF, ANA, and HLA-B27.

The case was considered FOT complicated by SAPHO syndrome. The patient was prescribed methotrexate 10 mg once weekly, minocycline and metronidazole orally, and compound sulfur lotion externally. At the 6-month follow-up visit, the skin rash and joint pain had improved.

### Case 3

A 42-year-old male presented with acne, head and axillary abscesses for more than 20 years, and anterior chest wall and lumbosacral pain for more than 10 years. In 1996, the patient noticed facial acne without pain or itching. Since 2002, the facial acne became more severe; examination revealed extensive areas of acne on the chest and back, and head and axillary abscesses were found along with local pain and itching. Additional pain in the chest wall and lumbosacral region also occurred. There was no fever, pus, or herpes on the hands and feet. The head abscess was incised and drained.

Laboratory tests showed elevated hsCRP (29.90 mg/L) and ESR (21 mm/h, reference <15 mm/h (14)); ANA, RF, and HLA-B27 were negative. Technetium-99 m-methylene diphosphonate (^99m^Tc-MDP) whole-body bone scintigraphy showed multiple areas of increased tracer uptake in the right shoulder joint, bilateral sternoclavicular joints, sternum, upper thoracic vertebrae, T11 vertebra, L3-5 vertebrae, upper end of the right femur, bilateral knee joints, and bilateral ankle joints. This led to the diagnosis of FOT combined with SAPHO syndrome.

The patient was treated with prednisone 25 mg once daily and minocycline 50 mg twice daily, and the glucocorticoid dose was gradually reduced to 12.5 mg once daily orally. At the 6-month follow-up visit, the patient reported relief of pain in the anterior chest wall and lumbosacral region and no new acne, but occasionally experienced left wrist pain.

### Literature review

FOT-associated spondyloarthritis has been reported in several case reports and small series. A total of 10 cases were identified from the English-language literature, which, together with our 3 cases, yielded a pooled cohort of 13 patients (Table 1). The patients included 12 males and 1 female, with a mean age of 35.6 years (range 17–50 years). They were of Asian (*n* = 3) or African American (*n* = 10) descent. All patients had peripheral or axial spondyloarthropathy. Skin lesions preceded the onset of spinal arthritis in all cases, with intervals ranging from 0.6 to over 15 years. The patterns of peripheral joint involvement were similar: asymmetric single or multiple arthritis involving large and small joints of the extremities (1, 7, 8, 15, 16). Axial symptoms (cervical and/or lumbar pain and stiffness) appeared in 6 patients (7, 15, 16). Only 2 patients had typical sternoclavicular involvement of SAPHO syndrome (1). None of the patients had dactylitis, psoriasis, uveitis, or inflammatory bowel disease. Imaging showed joint erosions in 6 patients, mainly involving the wrist, proximal interphalangeal, and metatarsophalangeal joints. Nine patients had imaging evidence of sacroiliac arthritis (6 bilateral, 3 unilateral). Laboratory examination showed elevated inflammatory markers and negative rheumatoid factor/HLA-B27 in all patients (1, 7, 8, 15, 16).

**Table 1:** A summary of clinical characteristics of FOT patients with spondyloarthritis.

Author, year	#1 our center 2022	#2 our center 2022	#3 our center 2022	#4 Rosner, 1982	#5 Rosner, 1982	#6 Rosner, 1982	#7 Ellis, 1987	#8 Ellis, 1987	#9 Ellis, 1987	#10 Ellis, 1987	#11 Libow, 1999	#12 Thein, 2004	#13 Lim, 2013
Age/sex	23, male	50, male	42, male	46, male	22, female	49, male	49, male	22, male	35, male	17, male	32, male	40, male	29, male
Race	Asian	Asian	Asian	African-American	African-American	African-American	African-American	African-American	African-American	African-American	African-American	African-Caribbean	African-American
Peripheral joint involvement	Shoulders, elbow, knee and ankle joints	Shoulders, elbows, knees and heels, and both hands and feet	Upper end of right femur, bilateral knee joints and bilateral ankle joints	Involved, not specified	Involved, not specified	Asymmetrically involved	Right foot, bilateral ankles, wrists and hands	Hip, feet	Fingers, 1st MTP, ankles	Fingers, toes, ankles	Left wrist, left 3rd PIP, Lateral epicondylitis	Both wrists, left ankle, Right elbow, both knees and shoulders	Both knees, ankles, feet or fingers
Axial involvement	Lumbar pain	Sternoclavicular joint,	Anterior chest wall and lumbosacral pain	Involved	Involved	Involved	Lower back pain	Low back, sternoclavicular, acromioclavicular joints	Neck and lower back pain	Neck and back pain	Cervical and lumbar	Left sacroiliac joint	Lower back pain
Therapy	Minocycline, cyproheptadine, folic acid with topical sulfamethoxate cream and polysulfide wash	Methotrexate, minocycline and metronidazole	Prednisone and minocycline	Not available	No data	No data	No data	No data	No data	No data	Isotretinoin, non-steroidal anti-inflammatory drugs	Prednisone and sulfasalazine	Adalimumab

Drug treatment was described in detail in 6 patients; all had tried NSAIDs, antibiotics, and topical medications with limited improvement. Prednisone was effective in 2 patients. One patient received adalimumab, achieving prolonged remission, while one patient responded satisfactorily to isotretinoin treatment (Table 2).

**Table 2:** Clinical details of patients with FOT complicated peripheral/axial spondyloarthritis involvement in our center.

Case	#1	#2	#3
Age/sex	23, male	50, male	42, male
hsCRP	64.17 mg/l	17.87 mg/l	29.90 mg/l
ESR	100 mm/h	22 mm/h	21 mm/h
HLA-B27	negative	negative	negative
Peripheral joint involvement	Bilateral shoulder, elbow, knee and ankle joints	Shoulders, elbows, knees and heels, and both hands and feet	Upper end of right femur, bilateral knee joints and bilateral ankle joints
Axial involvement	Lumbar pain	Sternoclavicular joint,	Anterior chest wall and lumbosacral pain
cutaneous manifestations	Facial papules/pustules/cysts; draining sinuses on scalp and axillae; truncal/limb exudate and papules; biopsy: hyperkeratosis, acanthosis, perifolliculitis, granulomas	Widespread acne/pustules/nodules/pitted scars (sparing hands/feet), worst on buttocks/axillae; biopsy: hyperkeratosis, dyskeratosis, acanthosis, neutrophilic infiltrates	Extensive acne on face, chest, back; head and axillary abscesses (incised/drained); no palmoplantar pustules
Skin biospy	Epidermal papilloma like hyperplasia, hyperkeratosis, acanthosis, and dermal collagen fiber hyperplasia.	Hyperkeratosis with dyskeratosis, thickening of the spinous layer, and infiltrating masses composed of neutrophils in the dermis	Not obtained
Increased radioactivity areas from ^99m^Tc-methylene diphosphonate (^99m^Tc-MDP) bone scintigraphy	Bilateral shoulder, elbow, knee and ankle joints	Bilateral sternoclavicular joints, first anterior rib, shoulder joint, pubic symphysis, knee joint and right heel	Right side shoulder joints, bilateral sternoclavicular joints, sternum, upper thoracic vertebrae, T11 vertebrae, L3-5 vertebrae, upper end of right femur, bilateral knee joints and bilateral ankle joints.
Therapy	Minocycline, cyproheptadine, folic acid with topical sulfamethoxate cream and polysulfide wash	methotrexate, minocycline and metronidazole	Prednisone, and minocycline

## Discussion

The central question of this study is whether follicular occlusion triad (FOT) with peripheral/axial spondyloarthritis (SpA) should be considered an analog of SAPHO syndrome or a distinct entity. Our case series and systematic review suggest that although FOT and SAPHO share overlapping cutaneous features—particularly hidradenitis suppurativa (HS) and acne conglobata (AC)—their musculoskeletal involvement patterns differ substantially. In our pooled cohort of 13 patients (3 from our center and 10 from literature), only 2 (15.4%) had typical sternoclavicular joint involvement, which is a hallmark of SAPHO syndrome, whereas whole-body MRI studies in SAPHO patients have reported anterior chest wall involvement in up to 53.6% (17). Furthermore, axial involvement in our FOT cohort predominantly affected the lumbar and cervical spine, while SAPHO commonly involves the thoracic spine (75% in one series (17)). These discrepancies suggest that FOT-associated SpA may not merely be a phenotypic variant of SAPHO but rather a separate disease pattern with a different pathophysiological background.

The histopathological features of FOT include perilobular inflammation of the reticular dermis extending subcutaneously, with hyperkeratosis, epithelial hyperplasia, perilobulitis, and follicular rupture. Over time, abscesses, sinuses, and fibrosis develop, leading to scarred lesions as seen in our patients (18). Although spinal involvement is rarely reported in FOT cohorts. For example, none among 66 Asian patients with dissecting cellulitis of the scalp (DCS)—epidemiological data indicate an increased risk of SpA in HS patients (19). A French prospective multicenter study found a 3.7% prevalence of spondyloarthropathy in HS patients, significantly higher than the 0.3% in the general population, and a US single-center study reported objective inflammatory arthropathy in 21 of 44 patients with HS and AC (16, 20).

Treatment options remain largely empirical. Conventional therapies include antibiotics, corticosteroids, zinc sulfate, isotretinoin, methotrexate, dapsone, and surgical interventions. In our patients, corticosteroids combined with minocycline provided satisfactory symptom control. However, biologic agents have emerged as a promising therapeutic strategy for refractory cases (7). Adalimumab, a TNF-α inhibitor, has been reported to achieve prolonged remission in FOT-associated arthritis. More recently, risankizumab, an IL-23 inhibitor, demonstrated efficacy in achieving both rheumatologic and dermatologic remission in a SAPHO patient with severe skin and joint involvement (21). Given the overlapping immune pathways between FOT and SAPHO, biologics targeting IL-17/IL-23 axis may offer a valuable option for patients with inadequate response to conventional disease-modifying antirheumatic drugs (DMARDs) (22–24). Nonetheless, controlled studies are lacking, and treatment decisions must be individualized.

Our study is limited by the small sample size inherent to the rarity of this condition. The relationship between FOT, SpA, and SAPHO remains incompletely defined. Nevertheless, our findings highlight that FOT-associated spondyloarthritis may represent a unique subset with distinct clinical and imaging features and potentially different treatment responses. Timely recognition by dermatologists and rheumatologists is crucial to prevent irreversible joint damage and disability. Larger prospective cohorts and mechanistic studies are needed to further clarify the nosology and optimal management of this underrecognized entity.

## Conclusion

FOT with peripheral/axial spondyloarthritis shows a unique disease pattern compared to SAPHO syndrome. As illustrated in our study, the arthritis usually develops insidiously and occurs months to years after the onset of the skin disease. Furthermore, testing for rheumatoid factor and HLA-B27 is typically negative in this group of patients. NSAIDs, oral and intra-articular corticosteroids, and DMARDs have all been used successfully; adalimumab has also been found to be effective. However, larger-scale controlled studies are needed to determine the most effective treatments for such patients.

## Data Availability

The original contributions presented in the study are included in the article/supplementary material, further inquiries can be directed to the corresponding author/s.
